# Covid‐19 Histamine theory: Why antihistamines should be incorporated as the basic component in Covid‐19 management?

**DOI:** 10.1002/hsr2.1109

**Published:** 2023-02-07

**Authors:** Harold L. Mashauri

**Affiliations:** ^1^ Department of Internal Medicine Kilimanjaro Christian Medical University College Moshi Tanzania; ^2^ Institute of Public Health Kilimanjaro Christian Medical University College Moshi Tanzania

**Keywords:** Covid‐19, healthcare management, infectious diseases, virology

## 1 INTRODUCTION

Since the emergency of Covid‐19 in December 2019 in Wuhan, China, WHO declared Covid‐19 to be a global pandemic crisis on March 11, 2020.^1^ The key in facing this outbreak lies greatly on proper understanding of its pathophysiology. A number of mechanisms have been proposed up to date with some supported clinical findings from the cases which has been successful treated.^2^ Some of these have determined the medical management of the disease in clinical settings despite some little progressive management plan fluctuations depending on the pathophysiology updates from recent studies. One among the suggested mechanisms was Covid‐19 Histamine theory.^3^ It explains the severity of the disease as the clinical consequences depends on the number of histamine mediated pathways activated in particular patient.

## 2 COVID‐19 AND HISTAMINE PATHWAYS

Covid‐19 Histamine theory explain clinical presentations of the disease as a result of Histamine activated pathways to a greater extent.^3^ It is basically activation of histamine pathways which lead to cytokine storm in Covid‐19 pathogenesis^4^ and other related complications. There are four classes of Histamine Receptors: H1, H2, H3, and H4.^5^ All of these plays a role in Histamine pathways. Pathologically, Histamine pathways has been found to significantly be able to modulate immune response and inflammation^6^ hence cytokine storm, tissue response to inflammation,^7^ coagulation process including deep vein thrombosis through Histamine H1 receptor^8^, ^9^ and can trigger acute symptoms due to its very rapid activity on vascular endothelium, bronchial and smooth muscles^10^ and so forth. All of these makes it one of the important cytokine for therapeutic target.

Of the most Histamine Receptors, H2 and H1 have gained the most clinical attention among researcher^11^, ^12^, ^13^ when comes to Covid‐19 pathogenesis. Although some experts argue that the use of antihistamines might interfere with the body first line defense mechanism against respiratory tract infections like corona virus,^14^ a number of Randomized Clinical trials and in vitro studies have shown several antihistamines to be of interest in management of Covid‐19 with good prognosis including reducing pulmonary symptoms. Some showed Famotidine, Cimetidine and cetirizine^11^, ^12^, ^15^, ^16^ while others has shown significantly antiviral effects from Hydroxyzine and possibly Azelastine which binds to ACE‐2 and Sigma‐1 receptor as off‐targets for viral attachment and replication^17^ even though the clear mechanism is not well known.

## 3 ROLE OF ANTIHISTAMINES

Taking into consideration Covid‐19 clinical presentation and its severity spectrum to death plus the clinical trials done already, histamine mediated pathways might be contributing to a great extent the prognosis of the Covid‐19 disease. Immunologically, it becomes so hard to point out quantitatively the role of every cytokine in the pathogenesis of the disease so as to know which cytokine specifically plays a major role. But at least from clinical presentation of a disease together with researched evidence, it shows histamines might hold a great deal hence antihistamines. Several studies have shown that a number of Covid‐19 patients improved significantly when on antihistamines due to their antiviral and anti‐inflammatory properties.^18^, ^19^, ^20^ Moreover, antihistamines have shown to be effective in the management of long term symptoms post‐Covid‐19 infection.^21^

Among all antihistamines, second generation seems to be of less side effects due to its specificity. Regarding whether Histamine H1 Receptor Antagonists can be used to in the management of Covid‐19 or not, some studies showed significant promise of the drugs as anti‐SARS‐Cov‐2 and immune‐modulating agent suitable for Covid‐19 treatment.^22^ H1 antihistamines have been used mostly to treat symptoms which are secondarily to histamine release. Some second generation antihistamines shows antiallergic and ant‐inflammatory effects which outlined in one study^23^ as through decrease in (1) production of cytokines by pro‐inflammatory drugs and in the release of other mediators by mastocytes and basophils, (2) recruitment of eosinophils in the late phase of allergic reactions, (3) expression of membrane receptors in nasal epithelia cells and the vascular endothelium, particularly the leukocyte Intercellular Adhesion Molecule 1 (ICAM‐1), which favors leukocyte migration form the blood to the respiratory mucosa and constitutes the main receptor for respiratory viruses to which the untreated atopic subject appear to be more susceptible.

Since antihistamines seems to hold a crucial prognostic role in the management of Covid‐19, there is a need to identify and repurpose some potential antihistamine drugs. One study suggested diphenhydramine, hydroxyzine, and azelastine to be considered in repositioning, then further research in them.^17^ Due to its potent, less side effects, rapid onset of action, specificity, antiallergic, and anti‐inflammatory properties,^24^ Cetirizine might be an important drug of consideration in managing Covid‐19 patients at the moment compared to other antihistamines or histamine receptors (H2, H3, and H4).

## 4 CONCLUSION AND RECOMMENDATION

Histamines pathways have been shown to mediate a number of clinical presentations including cytokine storm, runny nose, blood clotting issues, bronchoconstriction, lung‐hypersecretion and so forth, all of which have been presented with Covid‐19 patient. Its great role in modulating the immune response, viral viability, and influencing a number of systemic pathologies makes it an important target for the management of diseases like Covid‐19 which seems to trigger its pathway and bring a number of clinical complications.

From the above discussed findings on antihistamines and Covid‐19, specific antihistamines should be identified and included as a basic therapeutic approach toward Covid‐19 management plan along with other approaches. They seem to be promising in the management of Covid‐19 with short time of taking away the symptoms while giving the body enough time to reset its defense mechanism hence fast recovery. They work both by modulating the histamine pathways and suppressing viral growth. Despite the fact that still further clinical trials and studies should be done on the matter in identification and repositioning of potential antihistamines in the management of Covid‐19, there is no enough time for that while fighting this global health crisis. Selected antihistamines especially among Histamine H1 Receptor Antagonists should be approved for emergency use towards Covid‐19 management at the moment.

## AUTHOR CONTRIBUTIONS

**Harold L. Mashauri**: Conceptualization; data curation; project administration; resources; supervision; validation; visualization; writing—original draft; writing—review & editing.

## CONFLICT OF INTEREST STATEMENT

The author declares no conflict of interest.

## TRANSPARENCY STATEMENT

The lead author Harold L. Mashauri affirms that this manuscript is an honest, accurate, and transparent account of the study being reported; that no important aspects of the study have been omitted; and that any discrepancies from the study as planned (and, if relevant, registered) have been explained.

## Data Availability

Not applicable.
